# A study on the spatial and temporal evolution of urban shrinkage and its influencing factors from a multidimensional perspective: A case study of resource-based cities in China

**DOI:** 10.1371/journal.pone.0258524

**Published:** 2021-10-14

**Authors:** Ruili Wang, Chengxin Wang, Shuai Zhang, Xiaoming Ding

**Affiliations:** 1 College of Geography and Environment, Shandong Normal University, Jinan, China; 2 Communist Party China, Shandong Provincial Party School, Jinan, China; Institute for Advanced Sustainability Studies, GERMANY

## Abstract

With social and economic environment changes occurring in the world and deepening of the urbanization process, China’s urban development exhibits a new phenomenon of growth and shrinkage fluctuations. The resource-based city shrinkage phenomenon is particularly prominent. Research on the commonalities and patterns of similar groups should be enhanced. We constructed an urban shrinkage evaluation index system from the three dimensions of population, economy and space. Accordingly, we explored the spatiotemporal evolution characteristics of 175 resource-based cities in China from a multidimensional perspective with the entropy method, shrinkage model and transfer matrix method. The results indicated that most resource-based cities in China occurred in the non-shrinking state, but their development speed gradually decreased or even presented stagflation. The shrinkage measure-related results in the different dimensions revealed that the number of shrinking cities is increasing. The population, economic and comprehensive shrinkage levels were mainly slight and remained stable. The number of cities experiencing moderate and severe shrinkage was relatively small and mostly encompassed short-term shrinkage. Spatial shrinkage demonstrated a clear administrative hierarchy difference. Moreover, the spatial distribution range of shrinking cities in each dimension expanded and exhibited obviously similar characteristics, i.e., shrinking cities were relatively concentrated in Northeast China, while they were more scattered in other regions. Furthermore, the geodetector technique was applied to reveal the influencing factors of resource-based city growth and shrinkage. Based on the results, the change in the secondary industry output value share at the start of the study was the primary factor. The impact of each employment structure indicator from 2014 to 2018 was particularly significant. Comprehensive exploration of the shrinkage characteristics of this particular group of cities and their development behavior from a multidimensional perspective can provide an important reference for the transformation and high-quality development of resource-based cities.

## Introduction

Since the reform and opening up, the economy and cities in China have experienced continued growth and long-term rapid expansion. As economic development transitions into the new normal state and urbanization enters the second half of its course, the economic and urbanization growth rates gradually decrease, and urban development in China joins the postgrowth era [1]. Urban growth remains the mainstay of development. Certain cities, however, are beginning to experience local shrinkage centered on population decline. As a distinct group of problem cities in China, resource-based cities are subject to the inherent rules of resource industries and resource-based city development, and some cities are facing transformation and upgrading [2, 3], with the shrinkage phenomenon particularly prominent. Although there are differences in the definition, connotation, and motivation of shrinking cities across the academic community at present, resource-based cities in China experiencing population decline or economic recession due to industrial structure upgrading and population age structure changes exhibit mechanisms that are more similar to those pertaining to shrinking cities in certain developed countries [4]. To prevent China’s resource-based cities from experiencing the resource curse, we must comprehensively understand the current situation of urban growth and shrinkage and the influencing mechanism, improve the research system of different types of urban shrinkage, and provide new ideas for the formulation of resource-based city planning policies.

Urban shrinkage is not a new phenomenon. It initially originated from the notable population loss experienced by developed countries, but no clear shrinkage direction was provided in early studies. In the 1990s, Häußermann formally proposed the concept of shrinking cities in empirical research on the Ruhr area in Germany from a sociological perspective, which represented the demographic and economic decline conditions in cities in Germany due to deindustrialization [5]. Since then, the topic has gained increasing attention. Along with the global occurrence of urban shrinkage, scholars in different fields have carried out a series of studies on urban shrinkage in terms of the concept, connotation, quantitative identification, type and characteristics, motivational mechanisms, and response strategies. Theoretical and practical results have been obtained. Urban population decrease is an important definition aspect and measurement standard of urban shrinkage, which has been generally recognized [6, 7]. With the continued deepening of research, multidimensional observation perspectives such as economy, society, and space have gradually been added [8–10], further enriching the connotation of urban shrinkage. In the specific research process, quantification of a series of indicators from different perspectives has been incorporated into empirical evidence, which can be broadly classified into three categories: socioeconomic indicators, spatial statistical indicators and geographic landscape indicators [11, 12]. Socio-economic indicators, such as population change, industrial structure change, tax, unemployment rate and so on, are most widely used to identify shrinking cities [3, 13, 14]. Spatial statistics mainly measure the shrinkage level through the changes of indicators such as vacancy rate of urban buildings [15, 16] and infrastructure accessibility [15]. Geographic landscape statistics mainly use the theoretical knowledge of landscape ecology and remote sensing data to quantify urban shrinkage [17]. In addition, with the rise of urban big data, scholars have begun to explore its application in the field of urban shrinkage research. The nighttime light (NTL) data [13, 18] and Baidu map’s street landscape images [19] have been applied to identify shrinking cities. However, the definition of urban shrinkage has not yet established unified standards, so the selection of relevant measures reveals obvious differences. On the other side, previous studies have focused on the identification and determination of shrinking cities, but research on the process of urban growth and shrinkage from the track of urban development remains relatively insufficient. Thus, this paper brings growth and shrinkage into the research system at the same time, and analyzes the development of resource-based cities from a long time series, which is more in line with the volatility characteristics of urban shrinkage in China [10]. In recent years, the focus of empirical research has gradually shifted from shrinkage phenomena to motivational mechanisms, mainly focusing on the analysis of the causes of urban shrinkage in economic, social, resource and environmental dimensions [20–22]. The content has gradually been deepened, but research on the quantification of the motivation and evolution mechanism of urban shrinkage, remains relatively insufficient.

Under the background of globalization, the spatial distribution of shrinking cities is no longer limited to a few old industrial cities in developed countries represented by Britain and Germany [6], but gradually presents a global spread trend. The flow of capital around the world and technological innovation have accelerated the extraction of natural resources in some countries, leading to the economic and social recession in corresponding regions, especially in single industrial regions, such as resource-based cities or communities [10]. According to existing research, urban shrinkage caused by resource depletion, single industrial structure and deindustrialization has occurred in different countries under completely different development backgrounds, such as Detroit in the Midwestern USA, Halle in Germany, and old industrial areas in Russia, Australia, and China [18, 23]. The shrinkage of resource-based cities has gradually become prominent. According to statistics, the shrinkage proportion of resource-based cities was significantly higher than that of non-resource-based cities by 8.4% from 2011 to 2016 in China [14]. Northeast China, and China’s resource-exhausted cities experiencing prominent shrinkage or typical shrinking cities have always been the focus of urban shrinkage research [3, 24–26]. The shrinkage of small and medium-sized cities in Japan, especially resource-based cities, is also extremely prominent [9]. Six mining cities in Hokkaido, Japan (Yubari, Mikasa, Akabira, etc.) were in crisis of sharp population shrinkage of varying degrees [6]. There is also a relevant study taking the former mining city of Valley Jiului in Romania as an example [27]. Generally speaking, most researches on the shrinkage of resource-based cities tend to choose a smaller spatial scale. But there are few studies that have adopted the group of resource-based cities with similar development trajectories as research objects and have comprehensively measured these cities from different perspectives. Through the overall study of this special group, we hope to obtain the universal law of growth and shrinkage of resource-based cities in China.

To explore the evolution of shrinking resource-based cities, we considered 116 prefecture-level and 59 county-level resource-based cities as the research area. A comprehensive analysis of resource-based city shrinkage in China from 2010 to 2018 was conducted, and spatiotemporal evolution characteristics were analyzed. Then, with the application of the geodetector method, we explored the influencing factors within the context of the development characteristics of resource-based cities. This study aims to provide a reference for the transformation and development of shrinking resource-based cities, as well as other similar shrinking cities in China and abroad.

## Material and methods

### Study area

**Fig 1:**
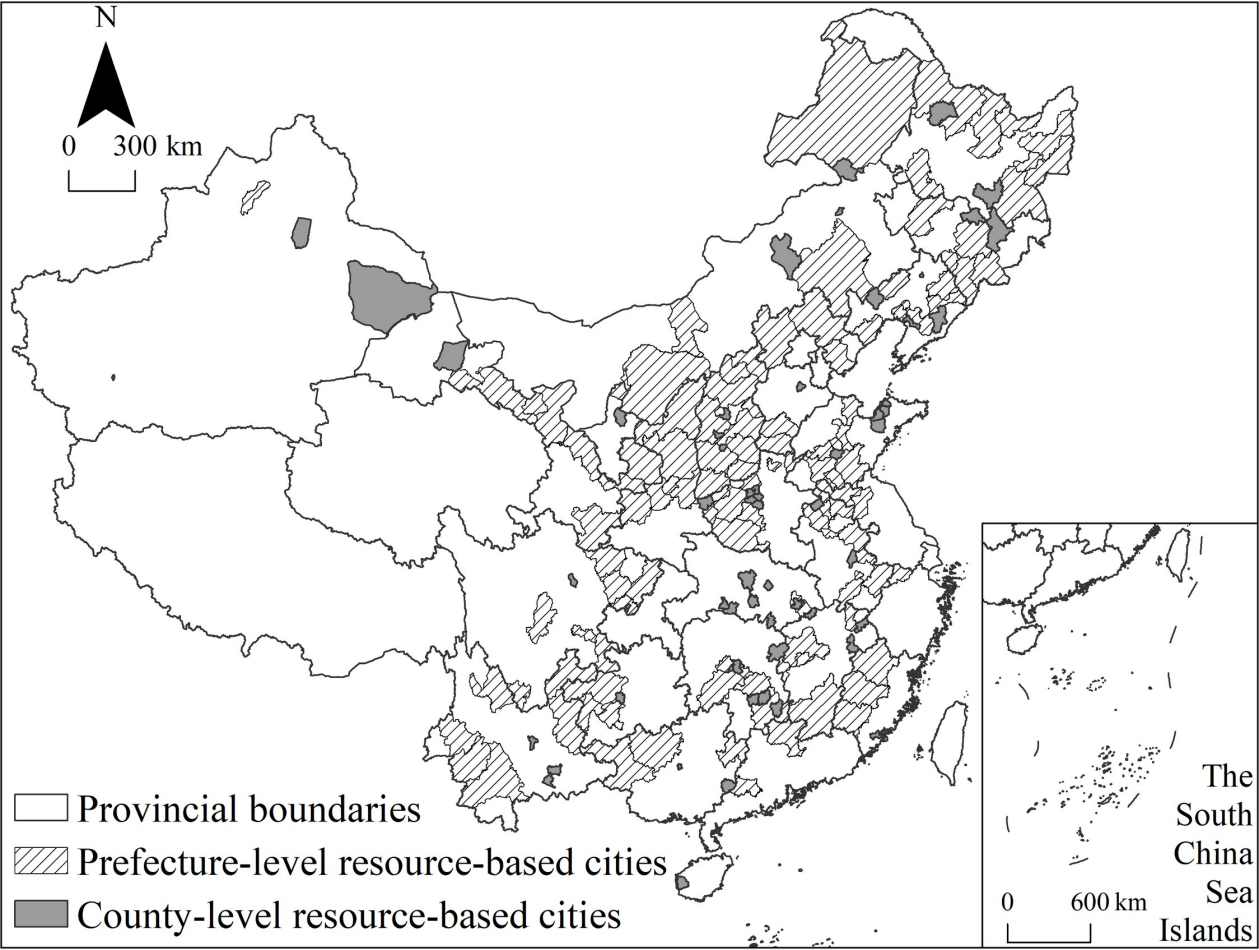
Study area. Note: The basic map came from a public map from the standard map service website of the Ministry of Natural Resources of China. The drawing approval number is GS (2016)1593.

According to the list of resource-based cities in China’s Sustainable Development Plan of National Resource-based Cities, 2013–2020 (SDPNRBC), which was issued by the State Council in November 2013, there are 262 resource-based cities nationwide. According to this list, there are 126 prefecture-level administrative regions, 62 county-level cities, 58 counties and 16 municipal districts. Since the research target encompasses the territory of urban entities, based on the existing data conditions, a total of 175 resource-based cities in 116 prefecture-level cities and 59 county-level cities were selected to minimize the differences in research unit data level. The study area is shown in Fig 1.

### Data source

Two types of data are mainly considered in this research: NTL data and socioeconomic statistics. NTL data comprise important monitoring data of the intensity of human activities, which reflect changes in urban expansion, social and economic activities, etc. These data facilitate the identification of urban spatial shrinkage and have been widely applied [13, 28–30]. The NTL data we adopted were obtained by the harmonized global NTL time series data [31]. We first converted the processed data into Lambert’s equal area projection, and extracted the NTL image of China in an ArcGIS environment. Then, it was resampled to raster data with a spatial resolution of 1km. Finally, the average NTL index was calculated for use. In addition, 18 additional socioeconomic statistics, including 5 indicators of urban growth and shrinkage and 13 influencing factors, were directly extracted from various statistical yearbooks or obtained after secondary calculations based on corresponding statistics. The study period was 2010–2018. In order to investigate the temporal variations, we divided it into two periods with 2014 as the node. Therefore, the data applied in this paper were relevant data in 2010, 2014 and 2018. The main statistical yearbooks we used included the China Urban Construction Statistical Yearbook 2010, 2014 and 2018, the China City Statistical Yearbook published in 2011, 2015 and 2019. The details are shown in Tables 1 and 4.

**Table 1:** The evaluation index system of resource-based cities’ growth and shrinkage in China.

Target layer	Criterion layer	Indicators	Data sources	Weights
**Comprehensive growth and shrinkage (CGS)**	PGS	Urban permanent residents	China Urban Construction Statistical Yearbook	0.2324
		Urban population density		0.1196
	SGS	Urban built-up area		0.2386
		Average NTL index	The harmonized global NTL time series data	0.1039
	EGS	Per capita GDP	China City Statistical Yearbook	0.1907
		Per capita fiscal revenue		0.1148

### Research methods

#### Measurement and classification of urban shrinkage

Against the new normal background, a single urban population index hardly fully characterizes the urban shrinkage phenomenon. However, it is easy to downplay its core features if numerous indicators, such as population, economy, society and spatial aspects, are all included in the conceptual system of shrinking cities [32]. Therefore, on the basis of fully understanding the connotation of urban shrinkage, this study built an index system (Table 1) around the three core elements of the population, economy and space and measured the growth and shrinkage levels of resource-based cities in China [13, 28, 33, 34]. Population shrinkage is the main characteristic of the urban shrinkage phenomenon [3, 25, 35]. The indicators of population growth and shrinkage (PGS) include the population size and population density, which are measured based on the total permanent population and the population density, respectively, in urban areas. The decline of population size is the most intuitive manifestation of urban shrinkage. Population density reflects the characteristics of population agglomeration. A decline in urban population density means that urban agglomeration economy is less likely to occur, and it is more prone to urban shrinkage [10, 14]. Economic shrinkage is also a remarkable manifestation of the urban shrinkage phenomenon. This paper selects the per capita gross domestic product (GDP) and per capita fiscal revenue to reflect the level of economic growth and shrinkage (EGS). Generally speaking, the higher the level of economic development, the less likely it is to experience urban shrinkage [14]. The spatial dimension is indispensable in urban shrinkage measurement. Due to the irreversible transformation of construction land, urban built-up areas impose limited effects on spatial vitality [28], but the corresponding growth rate can still reflect the vitality of urban spatial expansion. Therefore, we adopted the urban built-up area as one of the indicators and obtained NTL data for the evaluation of spatial growth and shrinkage (SGS). If the NTL index decreases, it indicates that the spatial vitality of the region is reduced and there may be an outflow of population and economic factors. In conclusion, all indicators are positive indicators, so there is no need for positive transformation. The weight of each index is obtained by entropy method, and the results are shown in Table 1.

Based on the obtained evaluation index data pertaining to the study period, comprehensive and fractal development index values for each city were calculated by using the weight of each index, and the growth and shrinkage levels of each city were calculated with a shrinkage model. The specific equation is as follows:

$$S_{a}=(P_{at_{2}}-P_{at_{1}})/P_{at_{1}}$$ (1)

Where *S*_*a*_ is the shrinkage degree of city *a*. $P_{at_{1}}$ and $P_{at_{2}}$ are the development indexes of city *a* in *t*_1_ and *t*_2_ respectively. That is, *S*_*a*_ is the change rate of the development index of city *a* from *t*_1_ to *t*_2_. *S*_*a*_ < 0 indicates that the city is shrinking, and the lower the value, the more serious the decline in the urban development level during the reporting period is over the initial period level, i.e., the higher the urban shrinkage level is. Conversely, city *a* occurs in a state of stagnation or growth, and the higher the *S*_*a*_ value, the greater the development level of city *a* is improved over the initial stage level.

Moreover, the classification of growth and shrinkage levels obviously cannot adopt a unified standard in different spatiotemporal scales. Therefore, the natural break point method is applied to our classification [14, 34]. Based on the data from 2010 to 2014, the cities occurring in the non-shrinking state were divided into two categories, namely, cities experiencing rapid growth and cities experiencing slow development. Shrinking cities were divided into three categories: slight shrinkage, moderate shrinkage and severe shrinkage. These cities were numbered as I, II, III, IV and V. The threshold ranges are listed in Table 2.

**Table 2:** Classification standard of urban growth and shrinkage.

Dimension	Severe shrinkage (V)	Moderate shrinkage (IV)	Slight shrinkage (III)	Slow development (II)	Rapid growth (I)
**CGS**	< -0.22	[-0.22, -0.11]	[-0.11, 0]	[0, 0.37]	≥0.37
**PGS**	< -0.50	[-0.50, -0.15]	[-0.15, 0]	[0, 0.35]	≥0.35
**EGS**	< -0.55	[-0.55, -0.23]	[-0.23, 0]	[0, 0.95]	≥0.95
**SGS**	< -0.41	[-0.41, -0.18]	[-0.18, 0]	[0, 0.86]	≥0.86

#### Transition matrix

In this paper, based on the classification of urban growth and shrinkage, the calculation idea of the land use type transfer matrix is adopted for reference [36, 37], the distribution of the urban development types of resource-based cities in different dimensions from 2010–2014 and 2014–2018 is superimposed and analyzed, and the urban type transfer matrix (Table 3) is constructed to explore the quantity distribution and transfer direction of growth and the shrinkage types of resource-based cities in China from a multidimensional perspective to reveal the dynamic change process of urban shrinkage. Among the various types, *L*_1_ to *L*_*n*_ denote urban growth or shrinkage types. *A*_*ij*_ indicates the number of cities that changed from type *i* into type *j* during the period from *T*_1_ to *T*_2_. $C_{{*}_{i}}$ denotes the total number of *i*-type cities during the *T*_1_ period, while $C_{j*}$ denotes the number of cities in the *j*-type development state during the *T*_2_ period. Moreover, $A_{ij}/C_{{*}_{i}}$ is the percentage of *i*-type cities converted from type *i* into type *j*.

**Table 3:** Transfer matrix of urban development types.

Types		*T*_2_						*nT*_1_
		*L*_1_	*L*_2_	⋯	*L*_*j*_	⋯	*L*_*n*_	
***T***_**1**_	***L***_**1**_	*A*_11_/C_*1_	*A*_11_/C_*1_	⋯	*A*_11_/C_*1_	⋯	*A*_11_/ C_*1_	C_*1_
	***L***_**2**_	*A*_21_/C_*2_	*A*_21_/C_*2_	⋯	*A*_21_/C_*2_	⋯	*A*_21_/C_*2_	C_*2_
	**⋯**	⋯	⋯	⋯	⋯	⋯	⋯	⋯
	***L***_***i***_	*A*_*i*1_/C_*i_	*A*_*i*2_/C_*i_	⋯	*A*_*ij*_/C_*i_	⋯	*A*_*in*_/C_*i_	C_*i_
	**⋯**	⋯	⋯	⋯	⋯	⋯		⋯
	***L***_***n***_	*A*_*n*1_/C_*n_	*A*_*n*2_/C_*n_	⋯	*A*_*nj*_/C_*n_	⋯	*A*_*nn*_/C_*n_	C_*n_
***nT***_**2**_		*C*_1*_	*C*_2*_	⋯	*C*_j*_	⋯	*C*_n*_	

#### Geodetector technique

The geodetector technique can reveal the internal and external influencing factors of geographical spatial differentiation through spatial variance analysis of statistical principles and has been widely applied in economic, social and ecological fields [38]. In this paper, the factor detection method in the geodetector technique is mainly employed to analyze the effect of each factor on the shrinkage of resource-based cities. The equation is as follows:

$$q=1-\frac{1}{N\sigma^{2}}\sum_{h=1}^{n}N_{h}\sigma_{h}^{2}$$ (2)

Where *q* denotes the influence of the detection factor, and the value occurs in [0, 1]. The larger the *q* value is, the greater the influence of a given detection factor on the dependent variable. *h* is the number of layers of the dependent or independent variable, and its value range is [1, *n*]. *N*_*h*_ and *N* are the number of units in the *h*-th layer and whole region, respectively, and $\sigma_{h}^{2}$ and *σ*^2^ are the variances in the dependent variables in the *h*-th layer and whole region, respectively.

The formation of urban shrinkage exhibits a profound local background, and the causes are extremely diverse. Considering the particularity of the development of resource-based cities, this paper implemented the comprehensive dimension growth and shrinkage degree (*Y*) as dependent variables and selected 13 indicators from 7 aspects, such as population growth, employment structure, and differences in people’s living conditions and regional economy, to constitute an index system of influencing factors regarding the shrinkage of resource-based cities (Table 4). Since the dependent variable is the change rate index of urban development, the influencing factor index was also defined as the index change rate over the corresponding time period. Moreover, to avoid considering the change value as an indicator, which could mask a negative population growth in certain cities, the natural population growth rate and population migration rate were both detected based on annual average values. With the help of the natural breakpoint method in the ArcGIS environment, each index data was divided into 8 grades to realize discrete processing and detect influencing factors. In view of the data availability, only 116 prefecture-level resource-based cities were chosen as experimental objects.

**Table 4:** The index system of influencing factors of resource-based cities’ growth and shrinkage.

Detection factors	Indicators	Data sources
**Population growth (POG)**	Natural population growth rate (*X*_1_)	China City Statistical Yearbook
	Population migration rate (*X*_2_)	Its original data comes from the Urban Construction Statistical Yearbook.
**Employment structure (EMS)**	Number of employees in the mining industry (*X*_3_)	China City Statistical Yearbook
	Number of employees in the manufacturing industry (*X*_4_)	
	Number of employees in the secondary industries/ Number of employees in the tertiary industries (*X*_5_)	
**Living conditions of people (LCP)**	Total retail sales of social consumer goods (*X*_6_)	
	Average salary (*X*_7_)	
**Regional economic differences (RED)**	GDP difference with provincial central cities (*X*_8_)	The original GDP data is from the China City Statistical Yearbook.
**Industrial structure (INS)**	Proportion of the secondary industry output to the GDP (*X*_9_)	China City Statistical Yearbook
	Proportion of the tertiary industry output to the GDP (*X*_10_)	
**Internal and external investments (IEI)**	Actual use of foreign investment (*X*_11_)	
	Gross fixed asset formation (*X*_12_)	
**Science and technology support (STS)**	Proportion of financial expenditure on science and technology (*X*_13_)	

## Results

### Identification results of urban shrinkage

According to the statistical results (Table 5), the mean of each dimension shrinkage of resource-based cities during the two periods always occurred within the slow development range. And the average of all dimensions from 2014–2018 declined to varying degrees over the initial period levels, while the coefficient of variation revealed an increasing trend, indicating that the development of resource-based cities in China generally occurred slowly and the development gap between cities widened. Based on the magnitude and proportion of growth and shrinkage of cities in the different dimensions, the non-shrinking cities still accounted for the majority in all dimensions, occupying more than 66.29% of all cities. However, compared to the initial stage of the study, the number of shrinking cities has significantly increased.

**Table 5:** Quantity statistics of multi-dimensional urban growth and shrinkage.

Period	Dimension	Mean	Coefficient of variation	Total number / proportion (%) of shrinking cities	Shrinking number/ proportion (%) at the prefecture level	Shrinking number /proportion (%) at the county levels	Total number /proportion (%) of non-shrinking cities
**2010–2014**	CGS	0.30	0.79	7/4.00	5/4.31	2/3.39	168/96.00
	PGS	0.12	1.79	33/18.86	20/17.24	13/22.03	142/81.14
	EGS	0.70	0.92	11/6.29	8/6.90	3/5.08	164/93.71
	SGS	0.54	1.01	21/12.00	8/6.90	13/22.03	154/88.00
**2014–2018**	CGS	0.17	0.84	53/30.29	10/8.62	43/72.88	122/69.71
	PGS	0.11	1.85	49/28.00	34/29.31	15/25.42	126/72.00
	EGS	0.69	1.41	27/15.43	15/12.93	12/20.34	148/84.57
	SGS	0.27	1.40	59/33.71	1/0.86	58/98.31	116/66.29

From 2010 to 2014, the population shrinkage-related characteristics of resource-based cities were particularly prominent, and the number of cities experiencing population shrinkage ranked first in all dimensions. The order of quantity distribution it presented from large to small is as follows: population shrinkage, spatial shrinkage, economic shrinkage, comprehensive shrinkage. From 2014 to 2018, the number and proportion of shrinking cities in all dimensions demonstrated a consistent growth trend. A sharp increase in spatial dimension shrinkage led to a notable increase in the number of cities experiencing comprehensive shrinkage during the same period, with the percentage rising from 4.00% to 30.29%. The quantity distribution order it finally exhibited from large to small is spatial shrinkage, comprehensive shrinkage, population shrinkage, economic shrinkage. By comparing the shrinkage degree of resource-based cities at the prefecture and county levels, it is found that the total number of shrinking cities at the prefecture level was slightly larger than that at the county levels in terms of the population and economic dimensions, while the shrinkage trend in the spatial dimension was the opposite.

### Transfer matrix for urban shrinkage

The transition matrix of the urban growth and shrinkage types is presented in Table 6, in which the urban development types are consistent with the grading results listed in Table 2. On the whole, the number of cities in the slow development interval (II) was much larger than the other types during the same period and dimension. Moreover, over time, the number of three types of shrinking cities notably increased, and the shrinkage range expanded. The shrinking cities in the population, economic and comprehensive dimensions exhibited the quantitative distribution characteristics of "the higher the shrinkage level, the less the quantity". It means that the shrinkage process of resource-based cities in China remained at the initial stage, with slight shrinkage as the main form. However, from the perspective of the spatial dimension, there were 34 cities experiencing severe shrinkage from 2014 to 2018, accounting for 57.63% of the total number of cities in the same dimension during the same period, and the proportion of cities experiencing moderate shrinkage was 30.51%. Compared to the initial stage of the study, the shrinkage quantity and overall shrinkage level were obviously improved.

**Table 6:** Transfer matrix of growth and shrinkage types in resource-based cities.

**CGS**		***T***_**2**_ **(%)**					***nT***_**1**_	**PGS**	***T***_**2**_ **(%)**					***nT***_**1**_
		**I**	**II**	**III**	**IV**	**V**			**I**	**II**	**III**	**IV**	**V**	
***T***_**1**_ **(%)**	**I**	36.92	32.31	13.85	10.77	6.15	65	**I**	5.88	76.48	11.76	5.88	0	17
	**II**	10.68	59.22	18.45	8.74	2.91	103	**II**	8.00	67.20	20.00	4.80	0	125
	**III**	0	66.67	33.33	0	0	3	**III**	7.41	44.44	44.44	3.70	0	27
	**IV**	33.33	33.33	33.33	0	0	3	**IV**	25.00	25.00	0	50.00	0	4
	**V**	0	100	0	0	0	1	**V**	50.00	50.00	0	0	0	2
***nT***_**2**_		36	86	30	16	7		***nT***_**2**_	15	111	39	10	0	
**EGS**		**I**	**II**	**III**	**IV**	**V**	***nT***_**1**_	**SGS**	**I**	**II**	**III**	**IV**	**V**	***nT***_**1**_
***T***_**1**_ **(%)**	**I**	32.73	58.18	5.45	3.64	0	55	**I**	2.50	60.00	0	2.50	35.00	40
	**II**	18.35	63.30	12.84	4.59	0.92	109	**II**	0	71.93	2.63	8.77	16.67	114
	**III**	60.00	20.00	20.0	0	0	5	**III**	0	25.00	16.7	58.33	0	12
	**IV**	0	100	0	0	0	3	**IV**	0	71.42	14.29	0	14.29	7
	**V**	0	66.67	0	33.33	0	3	**V**	0	50.00	50.00	0	0	2
***nT***_**2**_		41	107	18	8	1		***nT***_**2**_	1	115	7	18	34	

Through an analysis of the transformation of the urban development types, four conclusions can be drawn. Start by looking at the diagonal elements, only type II cities accounted for a relatively high proportion, at above 59%. It indicates that type II cities could maintain a low-speed or stagnant development state for a long time. Secondly, in the population, economy and comprehensive dimensions, the diagonal values of the type IV and V shrinkage cities were mostly 0. Notably, cities experiencing moderate and severe shrinkage were mostly subject to the short-term shrinkage phenomenon, so it was difficult to maintain the initial shrinkage level. Due to the large interval between the study periods, the shrinking cities in the three dimensions did not reveal obvious characteristics of adjacent grade transfer but tended to transform into lower-grade shrinking or non-shrinking cities. Furthermore, slightly shrinking (III) cities were more stable than were type IV and V cities, and the number and proportion of cities whose population dimensions slightly shrank were the highest in all dimensions. From the perspective of the transformation direction, the cities experiencing shrinkage in the population and comprehensive dimensions mainly transformed from type III into type II cities, revealing the characteristics of the coexistence of growth and shrinkage fluctuations. Cities subject to slight economic shrinkage were mainly transformed into type I cities. In contrast, the spatial dimension showed the reverse transformation characteristics, and the number of cities transformed from type III into type IV cities accounted for 58.33% of all cities, indicating that the spatial shrinkage level of resource-based cities greatly increased compared with the initial stage level. A final point to address here is, cities experiencing rapid growth (I) in all dimensions indicated an obvious deceleration or stagnation tendency, and their transformation direction was mainly from type I into type II cities. The proportion of cities with this transformation pattern in the population, economy and spatial dimensions reached 76.48%, 58.18% and 60.00%, respectively.

### Spatial distribution of multidimensional urban shrinkage

**Fig 2:**
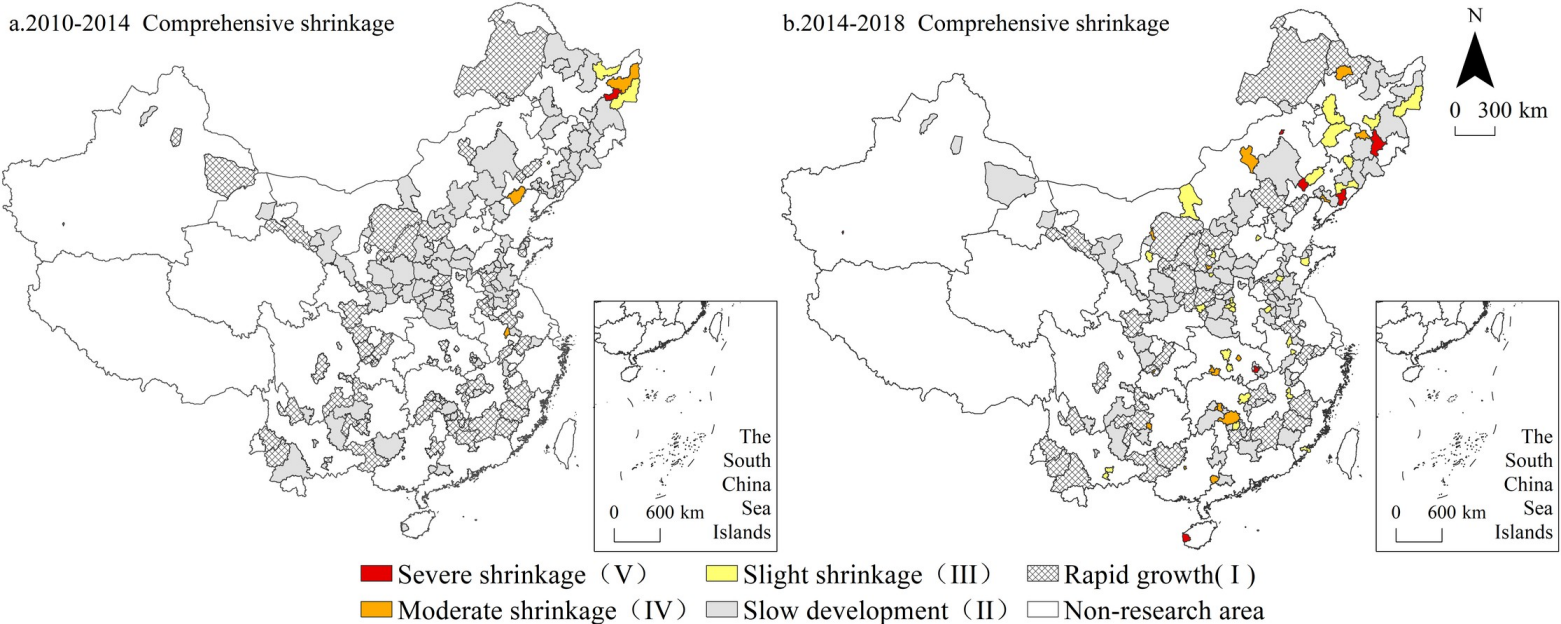
The spatial distribution of comprehensive shrinkage from 2010 to 2018. Note: The basic map came from a public map from the standard map service website of the Ministry of Natural Resources of China. The drawing approval number is GS (2016)1593.

The spatial distribution of comprehensive urban shrinkage (Fig 2) indicates that cities experiencing comprehensive shrinkage from 2010 to 2014 were mainly concentrated in the eastern part of Heilongjiang Province, including Qitaihe, Shuangyashan, Jixi and Hegang in Heilongjiang Province and Huludao and Diaobingshan cities in Liaoning Province, which are typical coal resource-based cities in Northeast China. From 2014 to 2018, the characteristics of comprehensive shrinkage became more prominent, and the number of cities transitioning from rapid growth and slow development to shrinkage increased notably, reaching 51, accounting for 30.36% of the total number of non-shrinking cities at the initial stage. Among these cities, due to spatial shrinkage enhancement, the shrinking cities at the county and city levels dramatically increased, accounting for 82.35% of all cities, which were scattered across all provinces. The shrinking cities at the prefecture level included Daqing, Songyuan, Liaoyuan, Fuxin, Benxi, Baotou, Wuhai and Hengyang, and the cities in Northeast China still comprised the majority.

**Fig 3:**
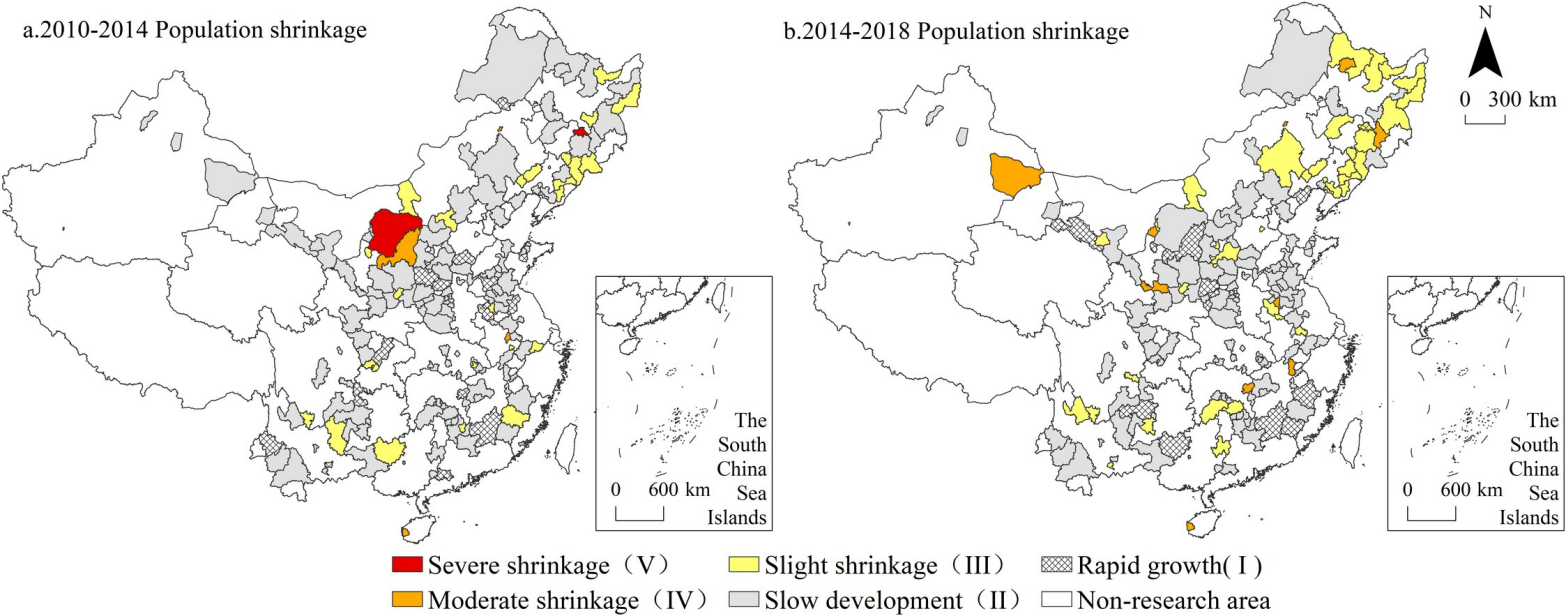
Spatial distribution of population shrinkage from 2010 to 2018. Note: The basic map came from a public map from the standard map service website of the Ministry of Natural Resources of China. The drawing approval number is GS (2016)1593.

As the core feature and the most intuitive expression of urban shrinkage, population shrinkage is more significant than are economic and spatial dimension shrinkage aspects (Fig 3). From 2010 to 2014, the cities experiencing population shrinkage still exhibited dispersion-oriented, small-scale agglomeration distribution characteristics, namely, two small-scale agglomeration areas were formed at the border of Jilin-Liaoning (Baishan, Tonghua, Liaoyuan, Benxi and Fengcheng) and the border of Ningshan-Inner Mongolia (Erdos, Yulin, Baotou and Lingwu), while other shrinking cities were scattered among the provincial marginal areas. From 2014 to 2018, Northeast China became the core area of resource-based cities with population shrinkage, forming a semicircular agglomeration area with slight shrinkage, including 21 cities comprising 3 type IV cities (Wudalianchi, Dunhua and Huolinguole) and 18 type III cities (Chifeng, Fuxin, Fushun, Yichun, Hegang, etc.), accounting for 42.86% of the total number of cities experiencing shrinkage during the same period.

**Fig 4:**
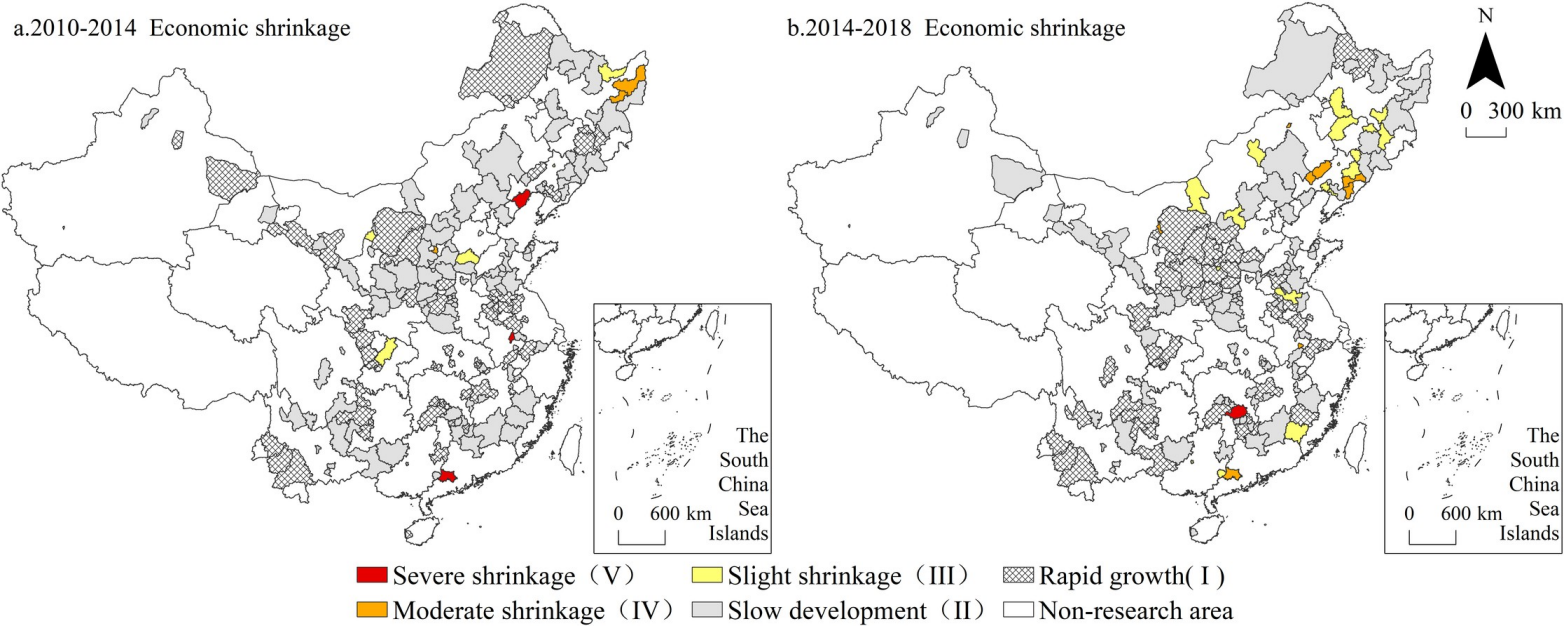
Spatial distribution of economic shrinkage from 2010 to 2018. Note: The basic map came from a public map from the standard map service website of the Ministry of Natural Resources of China. The drawing approval number is GS (2016)1593.

The spatial distribution of resource-based cities with economic shrinkage is shown in Fig 4. The economic development of China’s resource-based cities remains dominated by growth, and there is no large-scale and sustained economic recession. Compared to the population and spatial dimensions, the number of shrinking cities is obviously smaller, but this does not affect the distribution characteristics of small-scale agglomeration and multi-dispersion. From 2010 to 2014, those cities experiencing economic shrinkage formed the only agglomeration area in the eastern part of Heilongjiang Province, while the other cities were scattered. From the perspective of resource-based cities, 81.82% of these cities are coal resource-based cities, including the typical coal resource-based cities in Northeast China represented by Shuangyashan, Qitaihe and Hegang. Most of the cities with economic shrinkage from 2014 to 2018 were newly shrinking cities, and most of the cities with initial shrinkage entered the slow development zone during this period. During this period, the distribution of cities experiencing economic shrinkage moved southward, while Northeast China was also the focus area of economic shrinkage, with the percentage of shrinking cities reaching 55.55%. Liaoning Province became the province with the largest distribution of shrinking cities during the same period. From the perspective of the resource type, economic shrinkage was distributed among all resource-based cities, especially coal resource-based cities, including Wuhai, Fuxin, Beipiao and 12 other cities.

**Fig 5:**
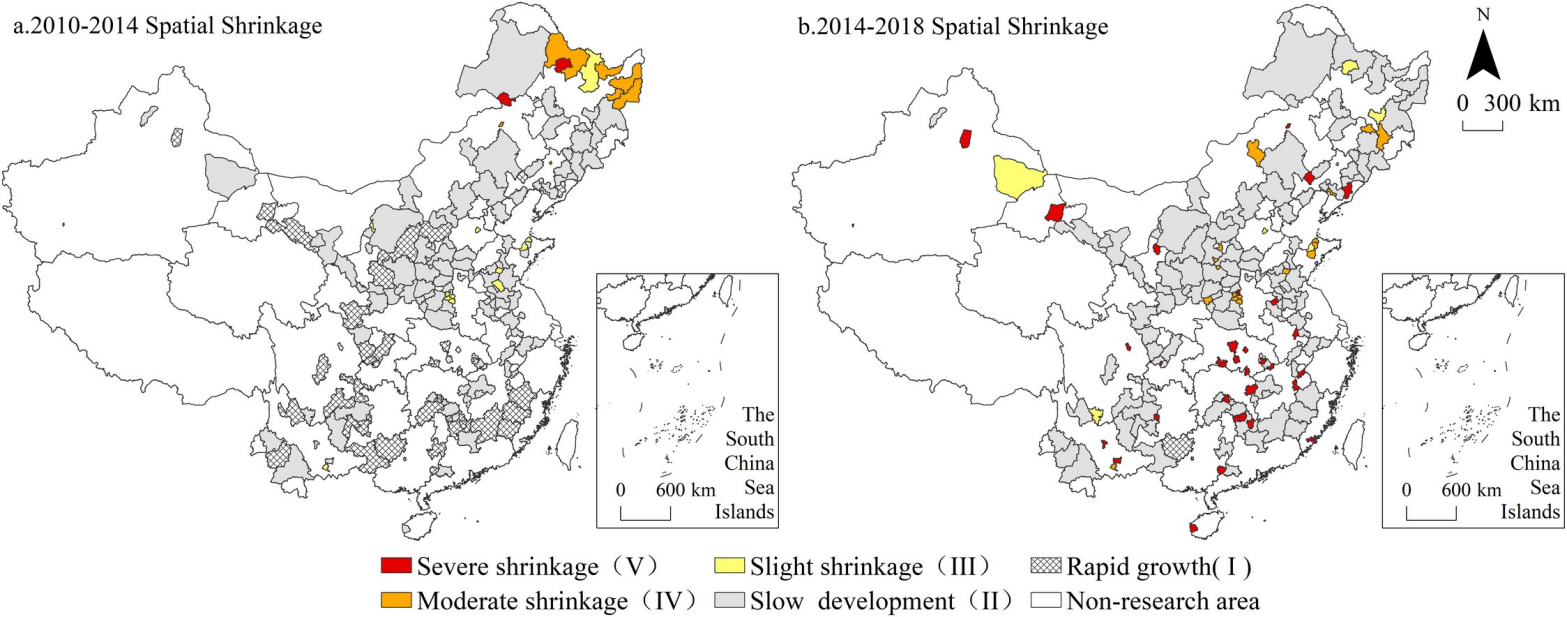
Spatial distribution of spatial shrinkage from 2010 to 2018. Note: The basic map came from a public map from the standard map service website of the Ministry of Natural Resources of China. The drawing approval number is GS (2016)1593.

The spatial pattern of urban spatial shrinkage is shown in Fig 5. From 2010 to 2014, cities experiencing spatial shrinkage were mainly concentrated in the marginal zone of Northeast China, such as Heihe, Wudalianchi, Yichun and Aershan in Inner Mongolia, and a few cities experiencing slight shrinkage were scattered across the Yellow River Basin. From 2014 to 2018, the number of cities experiencing spatial shrinkage increased greatly, and county-level shrinking cities dominated, accounting for 98.31% of the total number of shrinking cities. From the perspective of the spatial distribution, most of the cities with spatial shrinkage were located in the central region and revealed scattered distribution characteristics with a decreasing degree of shrinkage from southwest to northeast. Moreover, among the non-shrinking cities during the same period, the number of type II cities increased, thereby forming a continuous slow development zone.

### Empirical results of the influencing factors

We applied Geodetector software to identify the influencing factors and corresponding action intensity of the shrinkage phenomenon of China’s resource-based cities from 2010 to 2018. And the action directions of significant influencing factors were further analyzed in combination with the Pearson correlation coefficient. The results are shown in Table 7.

**Table 7:** Geodetection results of the influencing factors.

Detection factor	Indicators	*q* value and Pearson correlation coefficient	
		2010–2014	2014–2018
**POG**	*X*_1_	0.119	0.096
	*X*_2_	0.259** (0.318***)	0.086
**EMS**	*X*_3_	0.157* (-0.025)	0.146*(0.293***)
	*X*_4_	0.233***(-0.116)	0.203***(0.239***)
	*X*_5_	0.286***(0.120*)	0.240***(0.380***)
**LCP**	*X*_6_	0.149	0.113
	*X*_7_	0.233***(0.339***)	0.131
**RED**	*X*_8_	0.237***(-0.150*)	0.114**(-0.120*)
**INS**	*X*_9_	0.325***(0.519***)	0.117*(0.294**)
	*X*_10_	0.294***(-0.413***)	0.106
**IEI**	*X*_11_	0.168	0.142
	*X*_12_	0.316***(0.229***)	0.107**(0.146**)
**STS**	*X*_13_	0.223**(0.127*)	0.163*(0.266***)

**Notes:** *** indicates that the detection or correlation test is significant at the 0.01 level; ** and * indicate that the result passes the 5% and 10% significance test levels, respectively. A positive correlation suggests that higher index values correspond to higher shrinkage levels, and a negative correlation suggests that higher values indicate lower shrinkage levels. Additionally, to explore the direction of the indicators, we only test the correlation between indicators yielding significant geodetector results and the dependent variable. The results are listed in parentheses.

From 2010 to 2014, there were 10 indicators that impose significant effects on the spatial difference in growth and shrinkage levels between prefecture-level resource-based cities. According to the *q* value, the impact of indicators from large to small is *X*_9_, *X*_12_, *X*_10_, *X*_5_, *X*_2_, *X*_8_, *X*_7_, *X*_4_, *X*_13_, *X*_3_. According to correlation analysis, *X*_2_, *X*_5_, *X*_7_, *X*_9_, *X*_12_ and *X*_13_ have positive effects on the development of resource-based cities, while *X*_3_, *X*_8_ and *X*_10_ yield negative effects. However, *X*_3_ and *X*_4_ fail the correlation test. During this period, the change in the output value of the secondary industry was the primary factor influencing the growth and shrinkage levels of resource-based cities, i.e., the lack of development of resource-based industries was closely related to the emergence of the shrinkage phenomenon of resource-based cities.

With increasing regional differences in the growth and shrinkage levels of resource-based cities and with increasing scattered distribution degree, the index with a notable influence greatly decreased from 2014 to 2018, and only 7 elements such as the change in the number of employees in the mining and manufacturing industries exhibited significant detection results, but no new significant influence factors were observed. The influencing factors are sorted from large values as follows: *X*_5_, *X*_4_, *X*_13_, *X*_3_, *X*_9_, *X*_8_, *X*_12_. Overall, the factors of the employment structure imposed a particularly prominent influence on the shrinkage level of resource-based cities. During this period, except for a negative correlation between *X*_8_ and the dependent variable, all other indicators revealed positive effects. The influence of various factors and corresponding indicators on the shrinkage level of resource-based cities is examined in the fourth section.

## Discussion

### Spatiotemporal differentiation of resource-based city shrinkage

The economic development has entered a new normal in China. With an increasing demand for industrial structure transformation and upgrading and intensification of the contradictions between production cities, the shrinking characteristics of resource-based cities are gradually obvious, which is subject to their unique development path. Although growth remains the mainstream trend, the overall development speed is obviously decreasing, while the number and the shrinkage level of shrinking cities are increasing daily, and the situation is relatively pessimistic. It should be noted that type II cities can maintain low growth or stagnation for a long period, and these cities do not experience large-scale factor loss at the moment. However, in the urban development network, the regional growth advantage is reduced, and the region faces the risk of marginalization. Therefore, this kind of potential shrinking city should be given more attention over shrinking cities.

In additions, the results showed that relatively few cities could maintain shrinkage in both periods. This means, compared with the overall recession that is sometimes difficult to reverse in some developed countries, the shrinkage of resource-based cities in all dimensions exhibited significant instability. Then, from the long-term continuous series, are these cities still in continuous shrinkage, or do expansion and shrinkage exist at the same time? Urban shrinkage in this paper is calculated by the shrinkage model, which is lack of research on its sustainability. So, it should be further improved in the follow-up research.

### Influencing factors and countermeasures of urban shrinkage

The foregoing shows that there are many factors that significantly impact the development of China’s resource-based cities in different periods. Hence, in order to explore the sustainable development path of resource-based cities, we further explained the influence mechanism of main factors and put forward its countermeasures based on the existing research and our empirical results. In addition, we focus on the agglomeration area of shrinking cities, that is, Northeast China, and qualitatively explore the shrinkage incentives combined with the shrinkage characteristics of resource-based cities.

Among the resource-based cities, the proportion of cities experiencing a negative natural population growth represented by Northeast China was relatively small. It may be the main reason why *X*_1_ failed the significance test. Therefore, research on the influence mechanisms of the different dimensions and various scales should be strengthened. In contrast, population migration exerted a significant positive impact on the spatial difference between the growth and shrinkage levels of resource-based cities. This is similar to the conclusions of other studies [39, 40]. It indicates that the massive loss of population, especially the working-age population, aggravates the comprehensive shrinkage phenomenon of resource-based cities. To some extent, the empirical results of POG also suggest that the reason for the shrinkage of China’s resource-based cities is more likely to be the population out-migration under the action of economic and social factors than the change of population structure. Human resources, especially high-quality talent, are an important driving force of the economic transformation process of resource-based cities and regional high-quality development. Resource-based cities experiencing population shrinkage should urgently promote the construction of soft and hard environments, such as social, cultural and infrastructure environments, to enhance the attractiveness and cohesion of regional talent [41].

Based on the empirical results of the INS and EMS, it is found that resolution of the contradiction between industrial structure optimization and labor employment transfer is increasingly becoming a major problem faced by resource-based cities during critical periods. At the initial research stage, the scale of the emerging industries in resource-based cities was still small. As a result, regional resource elements were concentrated in traditional industries with a relatively high development level. Therefore, in a considerable number of cities, expansion of the traditional industrial scale and number of employees facilitates urban economic and social development to a certain extent, while the high-end manufacturing industry and tertiary industry are difficult to reap their economic benefits in the short term. At the latter period, further implementation of development planning of resource-based cities has effectively promoted the orderly development of alternative industries and consequent reduction in traditional resource-based industries. But the poor matching between the traditional industrial labor force and replacement industry [42] may lead to a large number of laid-off workers or high outflow from the region, which may cause fluctuations in short-term economic benefits and population and may increase the comprehensive shrinkage level of cities. This requires relevant cities to improve labor employment transfer conditions via the establishment of a more complete industrial system. Besides, this may better address the contradiction between sustainable industrial development and social employment in the future [43].

When exploring the causes of the decline of American urban centers, it was found that the location relative to larger urban centers was one of the basic factors that contributed to urban shrinkage [44]. It is similar to our results from the perspective of RED. We found that the greater the difference in development speed between cities and central cities, the more noticeable the shrinkage. Resource-based cities are far away from regional development centers. Under the condition that the actual distance is constant and the cost of time distance is shrinking, the capital, talents, information and other elements will accelerate to flow into the highland of economic development, with the gradual decline of the status of resource-based cities in the regional economic network [45]. In other words, the loss of regional competitiveness will compress their development space to a certain extent, resulting in more prominent regional shrinkage. For these cities, it is necessary to develop the upstream and downstream industrial chain and build a diversified industrial structure [23, 46], so as to improve the anti-risk ability and industrial competitiveness [40]. For another, STS yielded a positive effect on the development of resource-based cities during the study period. The relevant research shows that it is necessary to carry out strategic innovations to realize the successful economic transformation of resource-based cities [46]. Therefore, against the background of a deepening innovation-driven development strategy, appropriately increasing the scale of science and technology investments [28] and optimizing the structure of science and technology expenditures can effectively alleviate the problems of material capital extrusion and economic growth deceleration caused by long-term resource dependence, thus curbing its declining trend.

As a concentration area of urban shrinkage, Northeast China has attracted considerable attention. It is also impacted by many factors, experiencing both historical reasons and realistic causes. With reference to relevant literature, the following analysis can be seen here. First of all, earlier urbanization and the implementation of family planning have led to a lower overall fertility level from the perspective of population [47]. The research shows that the scale of migrant in Northeast China has been expanding, showing a state of population loss [48]. Meanwhile, the proportion of children in the region decreased, but the proportion of elderly people increased [49]. It means that the problem of fewer children and aging will be more serious in the future. Due to all these factors mentioning above, the population especially in resource-based cities in Northeast China, such as Hegang, Jixi and Fuxin, has experienced a negative growth for many years. In addition, the regional economic system, industrial structure, social policies and other comprehensive problems have also exacerbated its population shrinkage [39]. In regard to economic development, the shrinking cities in Northeast China are mainly coal resource-based cities. These cities achieved rapid economic and social development drawing support from resource advantages in the early stage. However, according to the development stage, most of them are in the mature or declining stage now [3], whose industrial structure is strongly solidified, and development of emerging industries is slow [50]. Obviously, the depletion of resources and the simplification of industrial structure have become the key factors of its contraction in the latter stage. Moreover, the government is one of the main forces that drive economic restructuring in resource-based cities [3]. Our results also confirmed that government internal investment had a significant inhibitory effect on the shrinkage of resource-based cities. Series plans for the revitalization of Northeast China, such as the Revitalization Plan of Northeastern China (2007) and the National Plan for the Adjustment and Transformation of Old Industrial Bases (2013) have helped speed up industrial restructuring [3]. But some studies have also pointed out that the newly created industries should be selected based on local industrial conditions and contexts. Otherwise, industries that rely solely on the introduction or construction of government investment may eventually decline because of weak industrial relevance or hinder the subsequent development [51].

## Conclusion

Resource-based cities are already located along the outer edges of metropolitan areas. In the new era of the overall economic slowdown, China’s resource-based cities, especially resource-exhausted cities, are experiencing or will soon face urban shrinkage represented by population loss and economic recession under the influence of multiple factors. This paper analyzed the spatiotemporal evolution of shrinkage and its influencing factors in 175 resource-based cities in China from 2010 to 2018. The study found that the development of China’s resource-based cities was still dominated by growth. However, the number of shrinking cities in the population, economy, and spatial dimensions increased. The spatial distribution range continued to expand. Certain areas in Northeast China became small clusters of shrinking cities. The industry and employment structures were the key factors influencing the growth and shrinkage levels of the resource-based cities in China during the two research periods. In summary, this study was conducted to explore the commonalities and differences between China’s resource-based cities from a multidimensional perspective, which perfected the framework for different types of urban shrinkage research to a certain extent. Drawing lessons from the idea of the land use transfer matrix, we employed the transfer matrix to describe the transformation characteristics of urban development. This method better highlights the direction of data change over other methods. Additionally, the combination of the geodetector technique and Pearson correlation coefficient analysis complemented the quantitative study on the factors of urban shrinkage.

It also should be pointed out that some deficiencies also exist in our research, which is worth studying in the future. Firstly, as mentioned earlier, our research on the sustainability of resource-based cities is relatively lacking. Secondly, the purpose of this study is to explore the law of the shrinkage of resource-based cities. But there are actually a few cities in the state of shrinkage according to the results. And the spatial distribution is very scattered, so it is relatively difficult to comprehensively summarize their distribution characteristics. From the comparison between resource-based cities and non-resource-based cities, we may get more reasonable results. Finally, we explored the influencing factors of the shrinkage of prefecture level resource-based cities from a macro perspective. However, quantitative research on local influencing factors of significant shrinking cities is still insufficient. It is also the focus of future research.

## Supporting information

S1 Data (ZIP)
